# Consumption of echium oil increases EPA and DPA in blood fractions more efficiently compared to linseed oil in humans

**DOI:** 10.1186/s12944-016-0199-2

**Published:** 2016-02-18

**Authors:** Katrin Kuhnt, Stefanie Weiß, Michael Kiehntopf, Gerhard Jahreis

**Affiliations:** Department of Nutritional Physiology, Institute of Nutrition, Friedrich Schiller University, Dornburger Straße 24, 07743 Jena, Germany; Institute of Clinical Chemistry and Laboratory Medicine, Jena University Hospital, Friedrich Schiller University, Erlanger Allee 101, 07747 Jena, Germany

**Keywords:** α-linolenic acid, Echium oil, Linseed oil, Stearidonic acid, Eicosapentaenoic acid, Metabolic syndrome

## Abstract

**Background:**

A plant-based strategy to improve long-chain (LC) omega (n)-3 PUFA supply in humans involves dietary supplementation with oils containing α-linolenic acid (ALA) alone or in combination with stearidonic acid (SDA). The study aimed to compare the effects of echium oil (EO) and linseed oil (LO) on LC n-3 PUFA accumulation in blood and on clinical markers.

**Methods:**

In two double-blind, parallel-arm, randomized controlled studies, all volunteers started with 17 g/d run-in oil (2 weeks). Thereafter, subjects received diets enriched in study 1 with EO (5 g ALA + 2 g SDA; n = 59) or in study 2 with LO (5 g ALA; n = 59) daily for 8 weeks. The smaller control groups received fish oil (FO; n = 19) or olive oil (OO; n = 18). Participants were instructed to restrict their dietary n-3 PUFA intake throughout the studies (e.g., no fish). To investigate the influence of age and BMI on the conversion of ALA and SDA as well as clinical markers, the subjects recruited for EO and LO treatment were divided into three subgroups (two age groups 20–35 y; 49–69 y with BMI 18–25 kg/m^2^ and one group with older, overweight subjects (age 49–69 y; BMI >25 kg/m^2^).

**Results:**

In plasma, red blood cells (RBC), and peripheral blood mononuclear cells (PBMC), EPA and docosapentaenoic acid (DPA) were ~25 % higher following EO compared to LO. Comparing all treatments, the effectiveness of increasing EPA and DPA in plasma, RBC, and PBMC was on average 100:25:10:0 and 100:50:25:0 for FO:EO:LO:OO, respectively. EO led to a lower arachidonic acid/EPA-ratio compared to LO in plasma, RBC, and PBMC. Following EO, final DHA was not greater compared to LO. Higher BMI correlated negatively with increases in plasma EPA and DPA after EO supplementation, but not after LO supplementation. Decreasing effect on plasma LDL-C and serum insulin was greater with EO than with LO.

**Conclusions:**

Daily intake of SDA-containing EO is a better supplement than LO for increasing EPA and DPA in blood. However, neither EO nor LO maintained blood DHA status in the absence of fish/seafood consumption.

**Trial registration:**

ClinicalTrials.gov Reg No. NCT01856179; ClinicalTrials.gov Reg No. NCT01317290.

## Background

A high concentration of long-chain (LC) n-3 PUFA in human tissue is associated with a lower risk of cardiovascular disease (CVD) [1, 2]. Thus, there has been an increasing interest in incorporating n-3 PUFA into the diet. In order to meet dietary eicosapentaenoic acid (20:5n-3; EPA) and docosahexaenoic acid (22:6n-3; DHA) recommendations, the American Heart Association recommends consumption of two servings of fish (particularly oily fish) per week [3]. However, the provision of marine LC n-3 PUFA for the (increasing) human population made difficult by problems such as the overfishing and pollution of the marine environment. Further, not everyone eats fish and some people suffer from fish protein allergy. Hence, there is a need to find alternative sources of LC n-3 PUFA for both human nutrition and aquaculture fish feed.

Linseed oil (*Linum usitatissimum* L.), also known as flaxseed oil, which naturally contains up to 60 % of the plant-based α-linolenic acid (18:3n-3, ALA), is the essential precursor of LC n-3 PUFA metabolites [4]. Supplementation with linseed oil has previously been shown to increase n-3 PUFA stores in humans. However, conversion of ALA to LC n-3 PUFA is limited and insufficient to achieve adequate tissue levels of EPA. DHA synthesis, in particular, is extremely limited [5–8].

Over the past few years, several novel dietary sources of LC n-3 PUFA for human consumption have been considered [9, 10]. High potential to improve LC n-3 PUFA supply has stearidonic acid (18:4n-3, SDA), another plant-based n-3 PUFA and an intermediate of ALA. SDA is present in high concentrations in some plant families, such as Primulaceae and especially Boraginaceae [11]. Seed oils from echium species (Boraginaceae) are unique due to their high concentrations of SDA and ALA together with γ-linolenic acid (18:3n-6, GLA) [11]. Oil from *Echium plantagineum* is an approved novel food available as a food ingredient. In humans, the ability of SDA to increase EPA in blood is higher than that of ALA, presumably because it bypasses the rate-limiting Δ6-desaturase step [12]. Transgenic SDA-containing soybean (16–28 % SDA), canola, and linseed oils have been developed [13, 14] and recently studied [14–17]. However, genetically modified foods are to date not well accepted by consumers in Europe [9]. Therefore, naturally occurring echium oil and linseed oil were chosen for the present project.

In general, SDA-containing oils possess potent anti-lipidemic and hepatoprotective effects. Echium oil has been shown to lower serum triglycerides [18–20]. Therefore, it is likely that echium oil may play a role in preventing progression of CVD and type 2 diabetes mellitus.

The primary aim of the entire project including the two present studies was to compare the increase of LC n-3 PUFA in plasma, RBC, and PBMC after consumption of 5 g ALA + 2 g SDA from natural echium oil (study 1) compared to only ALA (5 g) from linseed oil (study 2) under the same study conditions. The secondary aim was to investigate the influence of sex, increased age and higher BMI on conversion of ALA and SDA as well as on clinical blood markers. As controls for fatty acid metabolism, n-3 PUFA-poor olive oil (negative control) and EPA-fish oil (positive control) were supplemented throughout the entire project.

## Methods

### Ethics statement

The entire project including two randomized, double-blind, parallel-arm controlled studies were carried out in accordance with the Declaration of Helsinki of the World Medical Association, approved by the Ethics Committee of the Friedrich Schiller University Jena (No. 2270-04/08) and registered in the Clinical Trials Registry (ClinicalTrials.gov ID: NCT01856179 and NCT01317290). Written informed consent was obtained from all participants.

### Subjects

**Fig. 1:**
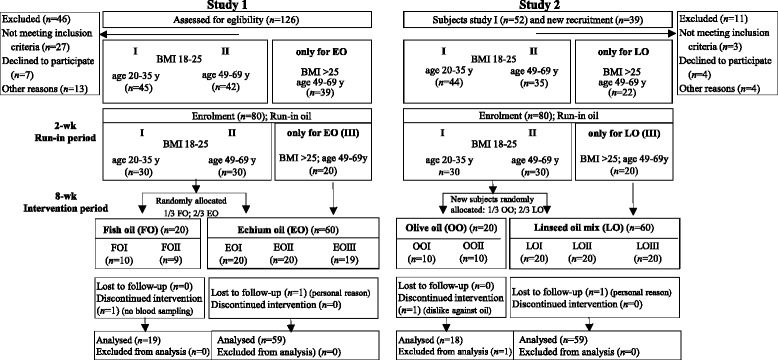
Flow diagram of subjects through study 1 and study 2. Subjects from study 1 who agreed to a repeat participation were implemented in study 2; former EO subjects were supplemented with LO in study 2; former FO subjects were supplemented with OO in study 2. EO, echium oil; FO, fish oil; LO, linseed oil mixture; OO, olive oil. Subgroup I, age 20–35 years and BMI 18–25 kg/m^2^; subgroup II, age 49–69 years and BMI 18–25 kg/m^2^; subgroup III, age 49–69 years and BMI >25 kg/m^2^

The recruitment for echium oil (EO; study 1, n = 60) and linseed oil (LO; study 2, n = 60) intervention was carried out according to age and BMI in three subgroups (subgroup I: BMI of 18–25 kg/m^2^, 20–35 years; subgroup II: BMI of 18–25 kg/m^2^, 49–69 years, n = 20; and subgroup III: overweight subjects, BMI > 25 kg/m^2^) (Fig. 1). Only normal-weight subjects in two age subgroups (20–35 y and 49–69 y) were recruited for the smaller control groups: fish oil (FO, positive control, study 1, n = 20) and olive oil (OO, negative control, study 2, n = 20). Gender was balanced in all groups. Recruitment of participants for the fish oil and echium oil interventions in study 1 (2011) has been described previously [20].

The subjects from study 1 were asked to participate in the subsequent study 2 (2012) to increase comparability. Former EO subjects were allocated to supplementation with LO, while former FO subjects were allocated to OO supplementation. In total, 80 % of the older subjects and 50 % of the younger subjects participated again in study 2. Exclusion criteria for both studies included: vegetarianism, veganism, daily alcohol abuse, pregnancy, lactation, chronic diseases, use of blood pressure-, cholesterol-, or TG-lowering medications or dietary supplements.

### Experimental design and diets

The two studies were performed according to same conditions (design, diets and analytical methods) as previously published [20, 21]. The same investigator and technical staff conducted both studies. Both studies started with a 2-weeks run-in period, in which all enrolled subjects (each n = 80) consumed 17 g/d of the same run-in oil. After that, subjects received the oil treatment for 8 weeks. In study 1, two thirds were randomly allocated to receive EO (n = 20 per subgroup) and one third was randomly allocated to FO (n = 10 per subgroup). In study 2, the newly recruited subjects were also randomly allocated to receive LO (n = 20 per subgroups I& II) and to OO (n = 10 per subgroups I&II).

Thus, in study 1 comprised EO (n = 60) and FO (n = 20) and in study 2 comprised LO (n = 60) and OO (n = 20) (Fig. 1). In order to reduce dietary intake of additional n-3 PUFA and linoleic acid (18:2n-6; LA) during the 10-week study, the participants were encouraged to avoid consuming the following foods: fish, fish oils, seafood, n-3 PUFA-rich foods, linseed and rapeseed oil, as well as margarine and common LA-rich sunflower oil. To reduce variation in dietary FA intake before blood sampling at day 0 and day 56, all volunteers received a defined diet for three successive days as described previously [20, 22].

### Supplemented oils

The run-in oil in both studies contained a mixture of fats and oils [20, 21] to represent the FA distribution of the Western diet. All four treatment groups received approximately 17 g/d of the respective study oil (Table 1). To balance the fat intake, also 17 g of run-in oil were supplemented in the first 2 weeks (run-in period) (Fig. 1; Table 1). The EO subjects received EO (seed oil of *E. plantagineum,* INCROMEGA V3, Croda; two tablespoons) containing an average of 5 g ALA, 2 g SDA and 2 g GLA (Table 1). The LO subjects were supplemented with 5 g ALA from linseed oil (Erfurter Ölmühle) [21]. The linseed oil was blended with common sunflower oil and a plant oil mixture (K-Classic) to obtain similar ALA dosage, n-6/n-3 ratio and LA content as in EO [16], (Table 1). The control groups received EPA-rich FO as positive control (1.9 g/d EPA; CRODA EPA-TG-500) or highly refined OO as n-3 PUFA-poor negative control (Gustav Heess). Female subjects received a slightly smaller quantity of study oil than male participants (15.5 g/d vs. 18.5 g/d) [20]; to provide comparable amounts of ALA (~2 % of total energy intake) for both genders. The handling of study oils and blinding was similar as previously stated [20, 21].

**Table 1:** FA profile and daily FA dose with run-in oil and with treatment oils^a^

	Run-in period (2 weeks)		Intervention period (8 weeks)							
Treatment	Run-in oil (*n* = 155)		Echium oil (study 1) (*n* = 59)		Linseed oil mixture (study 2) (*n* = 59)		Control groups			
							Fish oil^b^ (study 1) (*n* = 19)		Olive oil (study 2) (*n* = 18)	
	*%*	*g/d*	*%*	*g/d*	*%*	*g/d*	*%*	*g/d*	*%*	*g/d*
Oil dose		17		17		17		17		17
FA groups and individual FA										
∑SFA	44	7.5	11	1.8	9.0	1.4	12	2.1	14	2.1
∑MUFA (mainly 18:1n-9)	39	6.7	18	3.0	38	5.9	66	12	80	12
∑PUFA	16	2.8	70	12	53	8.1	22	3.3	6.0	0.9
18:2n-6, LA	16	2.8	17	2.9	22	3.3	5.7	0.9	5.5	0.8
18:3n-6, GLA	−	−	11	1.8	−	−	0.1	−	−	−
18:3n-3, ALA	0.2	−	30	5.0	31	4.9	0.6	0.1	0.5	0.1
18:4n-3, SDA	−	−	12	2.0	−	−	1.2	0.2	−	−
20:5n-3, EPA	−	−	−	−	−	−	11	1.9	−	−
22:5n-3, DPA	−	−	−	−	−	−	0.2	−	−	−
22:6n-3, DHA	−	−	−	−	−	−	1.4	0.2	−	−
n-6/n-3	80/1		0.7/1		0.7/1		0.4/1		11/1	

^a^Data are means of men and women. - indicates < 0.1 g/100 g or < 0.1 g/d. ^b^Fish oil (EPA:DHA, 9:1) blended with olive oil

### Blood sampling

Blood was collected at the Institute of Nutrition in Jena following the 2-weeks run-in period (day 0), and after 7 and 56 days of oil supplementation. Blood sampling methods and for preparation of blood fractions such as plasma, RBC, and peripheral blood mononuclear cells (PBMC) were described previously [20–22].

### Biochemical markers

Total cholesterol (TC), HDL-C, LDL-C, and TG (enzymatic colorimetric), oxLDL (ELISA), high sensitivity C-reactive protein and lipoprotein (a) (immunoturbidimetric), insulin (immunochemiluminometric) were analyzed according to the methods of the Institute of Clinical Chemistry and Laboratory Medicine at Jena University Hospital [20].

### Anthropometric parameters

Waist circumference, blood pressure, body composition (50 kHz-frequency impedance analyzer; Data Input) and body weight were recorded after the run-in period (day 0) and intervention period (day 56).

### Fatty acid analysis

Preparation of plasma, RBC, and PBMC fractions, and lipid extraction with chloroform/methanol/water (2:1:1, v:v:v) were performed as described by Kuhnt et al. [20–22]. In brief, FA were methylated with methanolic boron trifluoride into fatty acid methyl esters (FAME). Following purification by thin layer chromatography, FAME were analyzed by gas chromatography (GC) with flame ionization detector (60 m column length; DB225MS, Agilent Technologies). In all analyzed blood fractions, the same 47 FA were integrated (C10–C24). Individual FAME were expressed as a percentage of total identified FAME peak areas [% of total (Σ) FAME]. Samples were blinded during FA analysis. The reference standards used as FAME included: No. 463, 674 (Nu-Check Prep), BR2, BR4, ME93 (Larodan), Supelco®37 Component FAME Mix (Supelco) and PUFA No.3 (Matreya LLC). LabSolutions was used for GC peak integration (Shimadzu).

### Statistical analysis

All statistical analyses were performed using SPSS software 19.0. (IBM Corporation). The results are stated as means ± SD unless otherwise noted. The treatment effects on FA and biochemical markers were analyzed using the linear mixed model. Each variable was evaluated using analysis of covariance (ANCOVA) with sex, age, BMI, and the respective baseline value as covariates. Oil treatment as a factor for main effects and sex, age, and BMI as factors for interaction, were included in order to control type I. The differences between subgroup characteristics within and between treatments at baseline (day 0) were analyzed using subgroups as fixed factors without covariates (ANOVA). To examine the effect of time (day 0 to day 56) for total subjects of each treatment, repeated measures ANCOVA was used. In general, the *P* values of the pairwise comparisons were stated and were adjusted by using the step-down Bonferroni method. Pearson correlation analyses were conducted to test associations. Statistical significance was set at *P* ≤ 0.05 for all analyses.

## Results

### Characteristics of subjects

In the subgroups II and III with higher age and BMI, anthropometric measures and clinical markers were generally greater (except HDL-C) (Table 2). Weight gain, waist circumference, BMI, and portion of body fat did not differ between EO and LO treatments (data not shown).

**Table 2:** Characteristics of the study subgroups following the run-in period (day 0), prior to receiving echium oil or linseed oil mixture^1^

	Study 1 Echium oil			Study 2 Linseed oil mixture		
Subgroups	EOI (*n* = 20)	EOII (*n* = 20)	EOIII (*n* = 19)	LOI (n = 20)	LOII (*n* = 20)	LOIII (*n* = 19)
Sex, f/m	10/10	11/9	9/10	10/10	10/10	10/9
BMI group (kg/m^2^)	18–25	18–25	>25	18–25	18–25	>25
Age group (years)	20–35	49–69	49–69	20–35	49–69	49–69
Repeated participation (%)				40	90	80
Age (years)	28.1 ± 2.86^b^	58.8 ± 5.73^a^	61.2 ± 6.26^a^	25.0 ± 3.43^b^	59.0 ± 5.56^a^	61.1 ± 7.28^a^
Body mass index (kg/m^2^)	22.0 ± 2.34^b^	23.5 ± 2.40^b^	30.1 ± 3.30^a^	22.3 ± 2.24^b^	23.2 ± 2.27^b^	29.5 ± 3.26^a^
Waist circumference (cm)	80.3 ± 9.10^c^	87.2 ± 9.17^b^	104 ± 6.75^a‡^	80.7 ± 4.95^c^	86.3 ± 7.71^b^	99.4 ± 7.19^a^
Systolic BP (mm Hg)	125 ± 10.4^b*^	135 ± 19.9^ab^	141 ± 15.6^a‡^	133 ± 17.3^b^	148 ± 20.2^b^	154 ± 24.0^a^
Diastolic BP (mm Hg)	82.2 ± 8.9^b^	90.1 ± 12.3^a^	91.2 ± 7.90^a^	82.4 ± 6.68^a^	89.5 ± 10.7^a^	85.3 ± 19.8^a^
Total cholesterol (mmol/L)	4.52 ± 0.68^b^	5.82 ± 1.24^a^	6.30 ± 1.02^a^	4.43 ± 0.80^b^	5.58 ± 1.32^a^	5.84 ± 1.09^a^
HDL-C (mmol/L)	1.43 ± 0.30^a^	1.58 ± 0.47^a^	1.33 ± 0.32^a^	1.39 ± 0.27^a^	1.44 ± 0.38^a^	1.30 ± 0.33^a^
LDL-C (mmol/L)	2.54 ± 0.61^b^	3.54 ± 0.89^a^	3.95 ± 0.90^a^	2.48 ± 0.75^b^	3.36 ± 0.98^a^	3.70 ± 0.97^a^
LDL-C/HDL-C	1.87 ± 0.67^b^	2.37 ± 0.66^b^	3.08 ± 0.75^a^	1.86 ± 0.70^b^	2.42 ± 0.70^a^	2.90 ± 0.83^a^
Triglycerides (mmol/L)	0.89 ± 0.36^b^	1.14 ± 0.42^b^	1.82 ± 0.88^a‡^	0.97 ± 0.39^a^	1.31 ± 0.94^a^	1.39 ± 0.51^a^
Oxidized LDL (mmol/L)	67.7 ± 12.7^c*^	80.3 ± 11.4^b*^	92.9 ± 13.2^a*^	49.9 ± 18.0^b^	60.8 ± 15.2^a^	66.6 ± 14.2^a^
High sensitivity CRP (mg/L)	0.68 ± 0.62^a^	1.35 ± 2.03^a^	2.12 ± 2.41^a^	1.32 ± 1.60^a^	1.25 ± 1.50^a^	1.64 ± 1.43^a^
Insulin (mU/L)	6.03 ± 2.35^b^	9.75 ± 5.90^a^	11.6 ± 4.77^a^	6.22 ± 2.57^b^	7.96 ± 4.92^a^	10.8 ± 6.78^a^

^1^Values are means ± SD. *C* cholesterol. *EO* echium oil, *LO* linseed oil mixture; I, age 20–35 years and BMI 18–25 kg/m^2^; II, age 49–69 years and BMI 18–25 kg/m^2^; III, age 49–69 years and BMI >25 kg/m^2^. *P* values were analyzed using the generalized linear mixed model ANOVA

^abc^Within one oil treatment, means within a row with different superscript letters are significantly different between subgroups (*P* ≤ 0.05)

*Mean values are significantly different between the respective EO and LO subgroups (I, II, III) (*P ≤ 0.05, ^‡^ *P* 0.05 < 0.10)

### Fatty acid composition in blood fractions

#### Run-in period

Following the run-in period, of those who completed the study (*n* = 154), women had a lower plasma docosapentaenoic acid (22:5n-3, DPA) but a higher plasma DHA than men (*P* ≤ 0.001). Age was positively correlated with plasma EPA, DPA, and DHA (r = 0.538, 0.388, 0.279; *P* ≤ 0.001, respectively). BMI was also positively correlated with plasma EPA and DPA (r = 0.262, *P* ≤ 0.001; r = 0.172, *P* = 0.033) but not with plasma DHA (r = 0.12, *P* = 0.13).

#### Intervention period

Following 8 weeks of EO and LO supplementation, significant increases in ALA, SDA and the LC n-3 PUFA metabolites eicosatetraenoic acid (20:4n-3; ETA) EPA, and DPA were found in plasma, RBC, and PBMC (*P* < 0.01; Table 3). However, in all blood fractions the increase in ETA, EPA, and DPA were approximately 40–60 % higher with EO than with LO. In general, the greatest increase of ETA, EPA and DPA were found in plasma, followed by PBMC and RBC (Table 3). Final EPA and DPA in plasma, RBC, and PBMC were consistently higher (15–35 %) compared to LO, respectively (*P* < 0.05; Table 4). DHA did not accumulate with EO or LO and instead decreased in plasma, RBC, and PBMC; however, the decrease in plasma was lower with EO vs. LO (*P* = 0.008) (Table 3). The final plasma DHA was higher with EO compared to LO, but lower in PBMC (*P* < 0.01; Table 4).

**Table 3:** Percent change of FA in plasma, RBC, and PBMC in echium oil vs. linseed oil groups and in control groups after 8-weeks treatment^1^

Change of FA, %		Study 1 Echium oil (*n* = 59)	Study 2 Linseed oil mixture (*n* = 59)	Treatment effect EO vs. LO P^2^	Control groups	
					Fish oil (*n* = 19)	Olive oil (*n* = 18)
Plasma						
n-3 PUFA	ALA	230 ± 13^b†^	314 ± 20^a†^	*0.001*	−8 ± 4^c^	14 ± 5^c†^
	SDA	789 ± 69^a†^	159 ± 13^b†^	*<0.001*	59 ± 19^b†^	22 ± 14^b^
	ETA	406 ± 32^a†^	124 ± 10^b†^	*<0.001*	53 ± 12^b†^	27 ± 10^b†^
	EPA	168 ± 8^b†^	67 ± 6^c†^	*<0.001*	646 ± 75^a†^	4 ± 6^d^
	DPA	68 ± 4^b†^	31 ± 3^c†^	*<0.001*	113 ± 13^a†^	−1 ± 2^d^
	DHA	−3 ± 3^b^	−11 ± 2^b†^	*0.008*	39 ± 8^a†^	−5 ± 3^b^
n-6 PUFA	LA	−9 ± 1^b†^	3 ± 1^a†^	*<0.001*	−13 ± 2^b†^	−8 ± 1^b†^
	GLA	276 ± 23^a†^	−11 ± 3^b†^	*<0.001*	−16 ± 4^b†^	7 ± 6^b^
	DGLA	67 ± 5^a†^	−10 ± 2^bc†^	*<0.001*	−26 ± 4^c†^	6 ± 3^b^
	AA	14 ± 2^a†^	−1 ± 2^b^	*<0.001*	3 ± 3^ab^	0 ± 3^b^
	22:5n-6	−20 ± 3^b†^	−9 ± 3^b†^	*0.006*	−38 ± 7^b†^	12 ± 5^a^
RBC						
n-3 PUFA	ALA	156 ± 32^b†^	201 ± 9^a†^	*<0.001*	−12 ± 5^c^	8 ± 3^c†^
	SDA	405 ± 54^a†^	135 ± 22^b†^	*<0.001*	6 ± 13^c^	49 ± 35^b^
	ETA	243 ± 22^a†^	106 ± 7^b†^	*<0.001*	24 ± 6^c^	9 ± 4^c^
	EPA	85 ± 12^b†^	32 ± 4^c†^	*<0.001*	351 ± 35^a†^	−10 ± 3^d†^
	DPA	32 ± 8^b†^	12 ± 2^c†^	*<0.001*	75 ± 16^a†^	−4 ± 2^d^
	DHA	−4 ± 2^b†^	−10 ± 2^b†^	*0.15*	10 ± 9^a^	−9 ± 2^b†^
n-6 PUFA	LA	11 ± 3^a^	1 ± 1^b^	*<0.001*	−11 ± 4^b^	−4 ± 1^b†^
	GLA	144 ± 16^a†^	0 ± 4^b^	*<0.001*	1 ± 2^b^	7 ± 6^b^
	DGLA	48 ± 9^a†^	−5 ± 1^b†^	*<0.001*	−15 ± 3^b^	3 ± 1^b^
	AA	17 ± 3^a†^	−1 ± 1^b^	*<0.001*	2 ± 4^b^	0 ± 1^b^
	22:5n-6	−2 ± 4^a^	−4 ± 1^a†^	*0.43*	−29 ± 5^b†^	6 ± 2^a^
PBMC						
n-3 PUFA	ALA	147 ± 20^b†^	368 ± 28^a†^	*<0.001*	70 ± 43^bc^	13 ± 8^c^
	SDA	424 ± 78^a†^	103 ± 34^b^	*<0.001*	−2 ± 24^b^	47 ± 45^b^
	ETA	388 ± 35^a†^	81 ± 5^b†^	*<0.001*	38 ± 6^b†^	27 ± 19^b^
	EPA	97 ± 9^b†^	45 ± 5^c†^	*0.005*	564 ± 57^a†^	−1 ± 4^d^
	DPA	42 ± 5^b†^	27 ± 2^c†^	*0.006*	94 ± 8^a†^	3 ± 2^c^
	DHA	−20 ± 3^b†^	−15 ± 1^b†^	*0.12*	2 ± 5^a^	−4 ± 2^ab^
n-6 PUFA	LA	8 ± 2^a^	5 ± 1^a^	*0.42*	6 ± 3^a^	−7 ± 2^b†^
	GLA	128 ± 14^a†^	−1 ± 4^b^	*<0.001*	12 ± 15^b^	7 ± 4^b^
	DGLA	47 ± 17^a†^	−1 ± 1^b†^	*<0.001*	−15 ± 3^b†^	6 ± 2^b^
	AA	−2 ± 3^a^	−2 ± 1^a†^	*0.92*	−10 ± 2^b†^	2 ± 1^a^
	22:5n-6	−17 ± 6^a†^	−8 ± 2^a†^	*0.17*	−48 ± 3^b†^	8 ± 4^a^

^1^Values are means ± SEM; adjusted means not shown. Change is from day 0 to day 56. *EO* echium oil, *ETA* 20:4n-3, eicosatetraenoic acid, *LO* linseed oil mixture, *RBC* red blood cells, *PBMC* peripheral blood mononuclear cells. *P* values were analyzed using the generalized linear mixed model ANCOVA with sex, age, BMI, baseline value as covariates

^2^ *P* values for the treatment effect including only EO and LO groups

^abc^Means with different superscript letters are significantly different between all treatments (*P* ≤ 0.05)

^†^Within one oil treatment, means are significantly different between day 0 and day 56 (repeated measures; *P* ≤ 0.05)

**Table 4:** Final EPA, DPA, and DHA in plasma, RBC, and PBMC in echium oil vs. linseed oil groups and in control groups after 8-weeks treatment^1^

% of total FAME	Study 1 Echium oil (*n* = 59)	Study 2 Linseed oil mixture (*n* = 59)	Treatment effect EO vs. LO *P* ^2^	Control groups	
				Fish oil (*n* = 19)	Olive oil (*n* = 18)
Plasma					
EPA	1.63 ± 0.51^b^	1.07 ± 0.36^c^	*<0.001*	3.91 ± 0.99^a^	0.55 ± 0.20^d^
DPA	0.72 ± 0.14^b^	0.54 ± 0.11^c^	*<0.001*	0.84 ± 0.23^a^	0.43 ± 0.11^d^
DHA	1.49 ± 0.42^bd^	1.39 ± 0.34^c^	*0.009*	1.93 ± 0.55^a^	1.29 ± 0.39^cd^
RBC					
EPA	1.51 ± 0.33^b^	1.11 ± 0.31^c^	*0.001*	2.05 ± 0.28^a^	0.67 ± 0.23^d^
DPA	2.68 ± 0.38^ab^	2.44 ± 0.36^ab^	*0.049*	2.94 ± 0.37^a^	2.07 ± 0.33^c^
DHA	3.80 ± 0.83^ab^	3.62 ± 0.74^ab^	*0.35*	4.15 ± 0.73^a^	3.39 ± 0.89^b^
PBMC					
EPA	0.69 ± 0.21^b^	0.54 ± 0.14^c^	*<0.001*	1.91 ± 0.45^a^	0.30 ± 0.07^d^
DPA	2.37 ± 0.55^b^	2.11 ± 0.37^c^	*<0.001*	3.28 ± 0.38^a^	1.68 ± 0.23^d^
DHA	1.38 ± 0.39^d^	1.53 ± 0.34^b^	*0.003*	1.71 ± 0.28^ac^	1.51 ± 0.33^bc^

^1^Values are means ± SD; adjusted means are not shown. *EO*, echium oil, *LO* linseed oil mixture, *PBMC* peripheral blood mononuclear cells, *RBC* red blood cells. *P* values were analyzed using the generalized linear mixed model ANCOVA with sex, age, BMI, baseline value as covariates

^2^ *P* values for the treatment effect including only EO and LO groups

^abc^Means with different superscript letters are significantly different between all treatment groups (*P* ≤ 0.05)

Considering all treatments, increases in plasma, RBC, and PBMC of EPA were highest with FO (~520 %), followed by EO (~117 %), LO (~48 %), and OO (~0 %, *P* < 0.001; Table 3). The increase in plasma, RBC, and PBMC DPA was also significantly the highest with FO (94 %), but with a lower distance to EO (47 %), LO (23 %), and OO (0 %; Table 3). Overall, final EPA and DPA in plasma, RBC and PBMC were in similar order: FO > EO > LO > OO (*P* ≤ 0.001; not for DPA in RBC; Table 4). In plasma and PBMC, DHA was highest following FO, but was not different in RBC compared to EO or LO (Table 4).

**Fig. 2:**
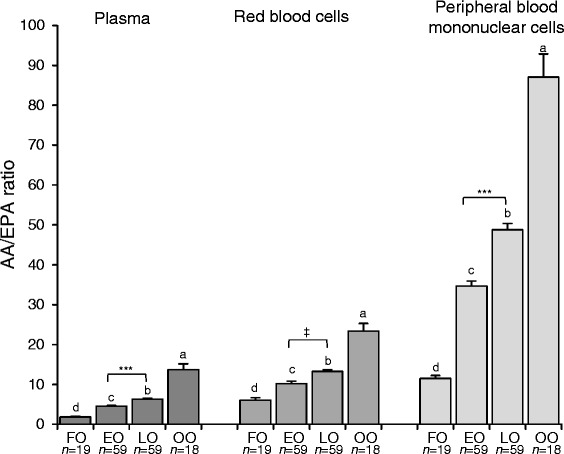
Final AA/EPA ratio in plasma, RBC, and PBMC in subjects following 8 weeks of fish oil, echium oil, linseed oil mixture, or olive oil. Values are means ± SEM. *P* values were analyzed using the generalized linear mixed model ANCOVA with sex, age, BMI, and baseline value as covariates. ^abc^Within one blood fraction, means with different superscript letters are significantly different between treatment groups (*P* ≤ 0.05). ***Means are significantly different between EO and LO treatment (*P* < 0.001; ^‡^ *P* 0.05 < 0.10). AA, arachidonic acid; EO, echium oil; FO, fish oil; LO, linseed oil; OO, olive oil

With respect to n-6 PUFA, EO supplementation increased GLA in plasma, RBC, and PBMC and was greatest compared to the remaining oil treatments (Table 3). In addition, DGLA and AA (the LC metabolites of GLA) also increased in all blood fractions, except for AA in PBMC (Table 4). Therefore, in all blood fractions, final GLA and DGLA were the highest with EO (*P* < 0.001; data not shown). The ratio of AA/EPA in plasma, RBC, and PBMC was lowest with FO compared to EO vs. LO vs. OO (*P* < 0.001; Fig. 2). The final AA/EPA ratio in plasma and PBMC was lower (*P* < 0.001) with EO vs. LO, but in RBC only as a trend (*P* = 0.09; Fig. 2).

In subjects consuming EO, BMI was negatively correlated with the relative increase in plasma EPA and DPA. In subjects consuming LO, there were no correlations to BMI in line with the non-interaction with BMI (data not shown).

### Effects on clinical markers

Correlations between age, BMI, anthropometrical and biochemical markers reflect differences in baseline characteristics between age and BMI subgroups (Table 2).

Change in HDL-C was clearly contrasting between EO and LO (Table 5), HDL-C decreased with EO, but increased with LO. Therefore, final HDL-C was lower with EO vs. LO (Table 5). The LDL-C decrease was greater with EO vs. LO. Finally, HDL-C/LDL-C ratio was lower with LO vs. EO. Plasma concentration of TC and TG decreased with EO, but final values did not differ between EO and LO (Table 5).

**Table 5:** Clinical markers in echium oil and linseed oil groups before and after 8-weeks treatment^1^

		Study 1 Echium oil (*n* = 59)		Study 2 Linseed oil mixture (*n* = 59)		Treatment effect EO vs. LO *P* ^2^
Total cholesterol (mmol/L)	Day 0	5.53 ± 1.25		5.27 ± 1.24		
	Day 56	5.22 ± 1.17	***	5.08 ± 1.20		0.59
HDL-C (mmol/L)	Day 0	1.45 ± 0.38		1.38 ± 0.33		
	Day 56	1.37 ± 0.42	***	1.58 ± 0.37	***	<0.001
LDL-C (mmol/L)	Day 0	3.33 ± 0.99		3.16 ± 1.03		
	Day 56	3.03 ± 0.87	***	3.05 ± 0.99		0.056
LDL-C/HDL-C	Day 0	2.43 ± 0.84		2.38 ± 0.84		
	Day 56	2.36 ± 0.88		2.00 ± 0.70	***	<0.001
Triglycerides (mmol/L)	Day 0	1.28 ± 0.70		1.22 ± 0.68		
	Day 56	1.13 ± 0.59	***	1.12 ± 0.62		0.66
Oxidized LDL (mmol/L)	Day 0	80.1 ± 16.0		58.9 ± 17.1		
	Day 56	75.8 ± 14.9	***	54.0 ± 17.7	*	<0.001
Insulin (mU/L)	Day 0	9.07 ± 5.07		8.26 ± 5.26		
	Day 56	6.25 ± 4.54	***	7.38 ± 3.69	*	0.011

^1^Values are means ± SD; adjusted means are not shown. *C* cholesterol, *EO* echium oil, *LO* linseed oil mixture. *P* values were analyzed using the generalized linear mixed model ANCOVA with sex, age, BMI, baseline value as covariates

^2^ *P* values for the treatment effect including only EO and LO groups

*Within one oil treatment, means are significantly different between day 0 and day 56 (repeated measures; ***P ≤ 0.001; *P ≤ 0.05)

In the control groups, FO showed the strongest TG reduction (−17 %), while TG increased with OO (+10 %; data not shown). The serum concentration of oxLDL decreased with both EO and LO; however, the significant difference between final oxLDL in the EO and LO groups (Table 5) was due to the significantly lower baseline values in the LO group (Table 2). The insulin concentration in serum decreased most with EO vs. LO (*P* < 0.001), while OO remained unchanged (Table 5).

## Discussion

Natural linseed oil contains a high portion of ALA (60–70 %) but little to no SDA [23]. In contrast, due to the high activity of Δ6-desaturase in *Echium* species, there are high levels of SDA (10–15 %) and GLA (8–12 %), the respective conversion products of ALA and LA [10, 11]. In the present study, the major aim was to investigate the effectiveness of SDA in EO compared to SDA-free LO for increasing endogenous LC n-3 PUFA stores in blood fractions under otherwise equal conditions (e.g. amounts of ALA and LA, n-6/n-3 ratio). Therefore, it was necessary to blend the LO with further plant oils. In addition, the habitual diet of participants contained no fish and seafood, which meant that there was minimal intake of dietary EPA and DHA.

### Increase in EPA and DPA

Eight weeks of EO supplementation led to a two- to three-fold EPA and DPA increase in plasma and cellular blood fractions compared to that achieved with LO. In one of the first clinical studies investigating SDA metabolism [12], similar quantities of SDA ethyl esters were compared with ALA (0.75 g and 1.5 g). The effectiveness of SDA in increasing EPA in plasma and RBC was about four-fold that of ALA [12]. In the present study, similar results were achieved using the natural complex oils (Tables 3 and 4). As the EO and LO supplements provided similar amounts of ALA (~5 g), it is likely that the additional 2 g of SDA provided by the EO was responsible for the additional increase in EPA and DPA.

The relative effectiveness of the four tested oils in increasing EPA and DPA in plasma, RBC, and PBMC was 100:25:10:0 and 100:50:25:0 for FO:EO:LO:OO, respectively. Similar results were obtained for the EPA increase in blood when purified ethyl esters of EPA, SDA, and ALA were administered to healthy male and postmenopausal female subjects (100:30:7) [12]. In contrast, in muscle tissue in lambs supplemented with EO or LO, only slight differences in LC n-3 PUFA were found [24]. As shown in Table 3, with respect to each oil treatment, the different intensities of increase in FA clearly represents the known metabolic pathway of n-3 and n-6 PUFA, as previously reviewed [25]. In general, the increases in EPA and DPA in RBC, considered as middle-to-long-term markers for FA intake and metabolism [26], confirmed that an 8-weeks EO or LO supplementation adequately achieved significant accumulation.

### No increase in DHA

Despite higher EPA and DPA in blood following EO supplementation, no consistently higher DHA could be observed compared to LO. In accordance with previous studies of EO, SDA ethyl esters, and SDA-enriched soybean oil, no increase in plasma, RBC, or PBMC DHA was found following 8 weeks of EO supplementation in this population [12, 15–18]. Previous clinical trials of ALA supplementation from LO (~5 g/d) [27–29] found increases in EPA (~50 %) with no change in DHA, similar to the effects achieved in our study. It is possible that conversion of DPA into DHA, which requires elongation, Δ6-desaturation, and a final chain shortening by peroxisomal β-oxidation, is limited by competition with dietary ALA [30]. We hypothesized that EO could compensate a DHA decline better than LO [20]. Surprisingly, the decrease in DHA was not significantly greater in the LO or OO groups compared to EO.

### Influencing factors on ALA and SDA conversion

The metabolism of ALA and also of SDA may be influenced by dietary factors, including amounts of ALA, LA, EPA, DHA, and the n-6/n-3-ratio [20, 28, 31–34]. Reducing the n-6/n-3 ratio in the diet may reduce the risk for CVD, but increasing the absolute amount of LC n-3 PUFA may be more effective [35]. In the present study, the EO and LO supplements provided similar n-6/n-3 and LA/ALA ratios, as well as absolute LA and ALA amounts. However, the EO did provide additional n-6 GLA, which may have led to greater competition for Δ5-desaturase and could decrease the efficacy of SDA conversion. Nonetheless, it is likely that the additional GLA was converted into DGLA, which has been also shown with Ahiflower oil™, an SDA- and GLA-containing plant oil from the Boraginaceae [36]. DGLA is metabolized to series-1 prostaglandins and thromboxanes that have potent anti-inflammatory and vasodilatory effects [25]. This may account, in part, for some of the beneficial effects of EO. AA, the direct product of DGLA, was only slightly increased. Furthermore, due to enhanced EPA accumulation, the AA/EPA ratio was lower in all blood fractions with EO compared with LO. A reduction in the AA/EPA ratio may have clinical relevance because AA-derived eicosanoids tend to be more pro-inflammatory and pro-aggregatory than eicosanoids derived from EPA [35]. It has been proposed that a 75 % reduction in plasma AA/EPA may impart clinically meaningful benefits [34]. In the present study, EO and LO supplementation resulted in 60–70 % and 40–50 % lower plasma and cellular AA/EPA ratio, respectively, compared to OO.

n-3 PUFA status is influenced by gender [37, 38], age [29, 39], and BMI [39]. In the current study populations, these factors appeared to influence baseline status, but have less effect on net increases of EPA and DPA during EO or LO consumption. In exception, during EO supplementation higher BMI was associated with lower increases of plasma EPA and DPA [20]. It may be possible that the expression and/or activity of enzymes necessary for SDA metabolism, such as elongases and Δ5-desaturase, are dependent on BMI. In support of this, a recent study comparing the effects of FO, SDA oil, and LO in obese and lean Zucker rats, genotype was a significant predictor for tissue FA composition following treatments [40].

### Clinical implications of EO supplementation

With respect to blood lipids/lipoproteins, EO supplementation resulted in more beneficial changes than LO, with the exception of HDL-C. In the majority of previous ALA-intervention studies (3–10 g/d; 6–12 weeks) [27–29, 41–44], no significant changes in plasma cholesterol fractions and TG were observed, despite increased plasma EPA. The known lowering of TG with FO [9] was confirmed with the present study (FO group). EO supplementation reduced TG independently of sex, age, and BMI [20]; however, these effects were not significantly greater than those achieved by LO supplementation. In rats, the TG and TC lowering effects of EO or SDA oil [19, 40] were explained by decreasing hepatic TG and TC and by downregulation of hepatic genes involved in FA and TG biosynthesis [19]. The cholesterol-lowering effect of EO could also be due to naturally occurring phytosterols (analyzed in *E. wildpretii* [45]), as is assumed for LO [29]. As a decrease in LDL-C and an increase in HDL-C is thought to reduce CVD risk, it is difficult to infer the clinical ramifications of the decrease in both LDL-C and HDL-C following EO supplementation. In contrast, LO supplementation resulted in an HDL-C increase. However, no other clinical studies of LO have found an increase in HDL-C [5, 29, 41, 42], except in non-smoking men [43]. Effects on oxLDL with supplementation of n-3 PUFA plant oils are rare. Compared to wheat, LO showed no change of oxLDL [44]. However, we found that both EO and LO reduced oxLDL compared to FO and OO controls.

Results from previous studies indicate that SDA may have novel therapeutic efficacy regarding the development of type 2 diabetes mellitus [46] and several obesity related pathologies [40]. In the present study, overweight adults with parameters of the metabolic syndrome responded to EO with a lower diastolic blood pressure and a reduction in blood lipids and serum insulin [20], which may reduce risk for CVD and type 2 diabetes mellitus [25, 46]. However, these effects were independent of age and BMI - healthy, normal-weight participants also benefitted from daily EO supplementation (e.g. decreased TG and insulin) [20]. It is widely accepted that EPA and DHA have beneficial effects on CVD [47]. However, the mechanisms by which ALA may exert beneficial health effects remain unclear [reviewed in 4]. To date, there is no high quality evidence to support a beneficial effect of ALA in reducing cardiac and sudden death [47]. Therefore, SDA-containing EO may be more cardioprotective than LO, which only provides ALA, due to greater conversion to EPA.

Limitations of the current project are that EO and LO treatments were investigated in two separate studies at different times (despite the same conditions) and the absence of a younger, overweight subgroup. Furthermore, the control groups, planned for FA comparison, were smaller than the EO and LO groups.

## Conclusions

In conclusion, 8 weeks of supplementation with EO produced significantly greater increases in plasma, RBC, and PBMC EPA and DPA, as well as a greater reduction in AA/EPA compared to LO. However, treatment with neither EO nor LO maintained blood DHA status in the absence of fish/seafood consumption. Furthermore, reductions in plasma LDL-C and serum insulin were greater with EO than with LO. Therefore, daily intake of SDA-containing EO is a better alternative than LO for increasing EPA and DPA in blood.

The studies were registered at www.clinicaltrials.gov as NCT01856179 and NCT0131729.

## Abbreviations

AA: arachidonic acid
ALA: α-linolenic acid
BP: blood pressure
CVD: cardiovascular disease
d: day
DGLA: dihomo-γ-linolenic acid
DPA: docosapentaenoic acid
EO: echium oil
FA: fatty acid
FO: fish oil
GLA: γ-linolenic acid
LA: linoleic acid
LC: long-chain
LO: linseed oil mixture
OO: olive oil
oxLDL: oxidized LDL
PBMC: peripheral blood mononuclear cells
SDA: stearidonic acid
subgroup I: age 20–35 y and BMI 18– 25 kg/m2
subgroup II: age 49–69 y and BMI 18–25 kg/m2
subgroup III: age 49–69 y and BMI > 25 kg/m2
TC: total cholesterol
